# Student–teacher relationships as a mediator of children's education‐linked genetics: results from the Norwegian Mother, Father and Child Cohort Study

**DOI:** 10.1111/jcpp.70179

**Published:** 2026-05-28

**Authors:** Chloe Austerberry, Elizabeth C. Corfield, Alexandra Havdahl, Dinka Smajlagic, Ragnhild Eek Brandlistuen, Mari Vaage Wang, Laurel Fish, Marialivia Bernardi, Jessica van de Grint‐Stoop, Mona Bekkhus, Pasco Fearon

**Affiliations:** ^1^ Centre for Child, Adolescent and Family Research University of Cambridge Cambridge UK; ^2^ PsychGen Centre for Genetic Epidemiology and Mental Health Norwegian Institute of Public Health Oslo Norway; ^3^ Psychiatric Genetic Epidemiology Group, Research Department Lovisenberg Diakonale Hospital Oslo Norway; ^4^ MRC Integrative Epidemiology Unit, Population Health Sciences, Bristol Medical School University of Bristol Bristol UK; ^5^ Department of Psychology, PROMENTA Research Center University of Oslo Oslo Norway; ^6^ Department of Child Health and Development Norwegian Institute of Public Health Oslo Norway; ^7^ Department of Education University of Oslo Oslo Norway; ^8^ Centre for Research on Special Needs Education and Inclusive Practice (SpedAims), University of Oslo Oslo Norway; ^9^ Research Department of Clinical, Educational and Health Psychology University College London London UK

**Keywords:** Behavioral genetics, Educational attainment, Teachers

## Abstract

**Background:**

Genetic differences are robustly associated with educational outcomes, but how they become linked is poorly understood. A plausible hypothesis, yet to be thoroughly empirically tested, is that the school environment mediates the association.

**Methods:**

Using structural equation models, we tested whether the student–teacher relationship at age 5 (teacher‐reported Student–Teacher Relationship Scale) mediated the association between children's education‐linked genetic propensities (PGS_edu_) and mother‐rated reading and math performance at ages 6–8. We performed these analyses in 63,032 children from the Norwegian Mother, Father and Child Cohort Study, a longitudinal, population‐based, pregnancy cohort.

**Results:**

Higher PGS_edu_ were significantly associated with lower student–teacher conflict (β = −0.08, *p* < .001) and positive teacher responses (β = 0.07, *p* = .001), but not student–teacher closeness (β = 0.01, *p* = .616). Associations between PGS_edu_ and educational performance were significantly partially mediated via conflict (β = 0.01, *p* < .001) and positive teacher responses (β = 0.01, *p* = .019) but not closeness (β = 1 × 10^−3^, *p* = .619).

**Conclusions:**

Student–teacher conflict and positive teacher responses may be mechanisms involved in the cascade of pathways linking children's genetics to their educational outcomes.

## Introduction

Educational outcomes are important in their own right and among the strongest predictors of many important outcomes, including economic success, physical and mental health, and longevity (Hout, 2012; Ross & Wu, 1995). Consequently, it is crucial to uncover the pathways through which these abilities develop. Individual differences in educational abilities (e.g., language, literacy, mathematics) and attainment (e.g., exam performance, years in education) are robustly associated with genetic differences. However, *how* these genetic differences translate into differences in educational outcomes is poorly understood. Gene–environment correlation (*r*GE)—occurring when genetic differences are associated with environmental exposure—has long been speculated to play an important role (Dickens & Flynn, 2001; Plomin, DeFries, & Loehlin, 1977; Scarr & McCartney, 1983), and evidence suggests that children's social environments are a highly promising mechanism to investigate. Specifically, children's genetically influenced traits may evoke social responses that support or hinder educational development. Most existing research has focused on genetically evoked responses from parents (Austerberry et al., 2024, 2025; Tucker‐Drob & Harden, 2012; Wertz et al., 2020). However, despite children typically spending much of their time in school—an environment specifically dedicated to promoting educational outcomes—the school social environment has been critically overlooked as a potential mediator of genetic effects on educational success. The present analyses addressed this gap by examining whether the student–teacher relationship acts as a gene–environment pathway and mediates associations between children's education‐linked genetic propensities and their later educational outcomes.

Heritability estimates the proportion of phenotypic variation attributable to genetic differences. Large‐scale meta‐analyses have found language skills to be ~24% heritable in infancy (Austerberry, Mateen, Fearon, & Ronald, 2022), language and educational skills 34%–80% heritable in middle childhood (Andreola et al., 2021; de Zeeuw, de Geus, & Boomsma, 2015), and educational attainment to be 41%–46% heritable in adulthood (Silventoinen et al., 2020). There is also robust evidence of a linear increase in general intelligence heritability from ~23% in early childhood (Davis, Haworth, & Plomin, 2009) to 41% in middle childhood (Haworth et al., 2010), 55%–66% in adolescence (Haworth et al., 2010), and 62%–81% in adulthood (Lee, Henry, Trollor, & Sachdev, 2010). A convincing hypothesis to explain this increase is that individuals with certain genetic predispositions seek out particular environments (active *r*GE) or elicit specific responses from others (evocative *r*GE), reinforcing their predispositions. It is theorized that over time these choices and responses further align the individual's environment with their genetically influenced traits, amplifying heritability estimates (Dickens & Flynn, 2001; Plomin et al., 1977; Scarr & McCartney, 1983).

There is growing evidence that children's genetically influenced traits evoke responses from caregivers (Avinun & Knafo, 2014; Kendler & Baker, 2007). This includes evidence suggesting children's cognition‐ and education‐linked genetics evoke differences in parental warmth and cognitive stimulation (Austerberry et al., 2025; Austerberry, Fearon, et al., 2024; Tucker‐Drob & Harden, 2012; Wertz et al., 2020). These evoked differences may also mediate genetic effects on cognitive and educational performance (Austerberry et al., 2025), although the evidence is somewhat mixed (Austerberry, Fearon, et al., 2024; Zhou et al., 2024). One limitation of this literature is that it has tended to focus only on whether children's genetics evoke responses from parents, neglecting the possibility that the school environment (e.g., student–teacher relationship) might also play a role in mediating the effects of genetic propensities on educational outcomes. Additionally, studies of biological parents and their offspring face a fundamental challenge—because children share much of their genetic material with their parents, it is difficult to separate evocative effects of the child's genetics on parenting from the parents' own genetics. Because teachers and children are genetically unrelated, associations between child education polygenic scores (PGS_edu_) and the student–teacher relationship cannot reflect shared genetics, reducing a key source of confounding common in *r*GE studies of parents, offering a cleaner test of evocative effects. However, indirect parental genetic effects (Demange et al., 2022; Kong et al., 2018) and SES‐linked environmental pathways—for example, child SES influencing teacher perceptions of students (Doyle, Easterbrook, & Harris, 2023; Rist, 2000)—may still contribute to observed associations.

There is good reason to believe the student–teacher relationship is a mechanism worth investigating. Non‐genetic research suggests children's academic skills influence the student–teacher relationship, with a meta‐analysis of 19 studies finding less conflict and greater closeness among higher performing, more motivated, and engaged students (Nurmi, 2012). Furthermore, extensive educational psychology research indicates positive student–teacher relationships are associated with better cognitive and educational outcomes (Roorda, Koomen, Spilt, & Oort, 2011; Tao et al., 2022; Vandenbroucke, Spilt, Verschueren, Piccinin, & Baeyens, 2017). Consistent with this, research from the Norwegian Mother Father and Child Cohort Study (MoBa) found student–teacher closeness in kindergarten was associated with better academic and behavioral outcomes across diverse risk groups (Haag et al., 2025), potentially buffering risk by supporting school readiness (Baardstu, Wang, & Brandlistuen, 2022). However, little is known about whether children's education‐associated genetic propensities evoke differences in these relationships, or whether the school environment mediates the association between children's genetics and their educational outcomes.

We addressed this gap by testing the following three preregistered hypotheses: (Austerberry et al., 2024)

1. Children's PGS_edu_—which measure genetic propensity for educational attainment—are positively associated with closeness and reduced conflict in the student–teacher relationship and positivity of teacher responses to students at age 5, the latter designed to capture teacher responses within the dyad more directly than the measures of closeness and conflict, which also refer to child behavior.
2. Closeness, reduced conflict, and positivity of teacher responses to students at age 5 are positively associated with educational performance at ages 6–8.
3. Closeness, reduced conflict, and positivity of teacher responses at age 5 mediate the association between PGS_edu_ and educational performance at ages 6–8.

## Methods

### Sample

We used data from the Norwegian Mother, Father and Child Cohort Study (MoBa), a population‐based pregnancy cohort conducted by the Norwegian Institute of Public Health. Participants were recruited from across Norway in 1999–2008. Women consented to participation in 41% of pregnancies (*N* = 112,908). Comparisons with the national Medical Birth Registry of Norway indicate that although participants differ somewhat from the general birthing population (e.g., younger mothers, smokers, and single mothers are under‐represented), estimates of exposure–outcome associations appear largely unbiased by self‐selection (Nilsen et al., 2009). The cohort includes approximately 114,500 children, 95,200 mothers, and 75,200 fathers. The establishment of MoBa and initial data collection were based on a license from the Norwegian Data Protection Agency and approval from the Regional Committees for Medical and Health Research Ethics (REK). The MoBa cohort is currently regulated by the Norwegian Health Registry Act. The present study was approved by the REK (21076). Blood samples were collected from both parents during pregnancy and from children (umbilical cord) at birth (Paltiel et al., 2014). Further information on recruitment and data collection is reported in published cohort profiles (Brandlistuen et al., 2025; Magnus et al., 2006, 2016). Protocols, including consent forms and questionnaires, are published elsewhere (Norwegian Institute of Public Health, 2019). Some of the analyzed data were collected from childcare centers by the Language and Learning Study (SOL), a MoBa sub‐study conducted in collaboration with the Norwegian Institute of Public Health and the Ministry of Education (Wang & Schjølberg, 2014). Questionnaires to childcare centers were administered only to the later MoBa birth cohorts (born in 2007–2009), resulting in this data being available for a subset of participants (see Table 1).

**Table 1:** Means, standard deviations, and bivariate correlations between genetic and phenotypic variables

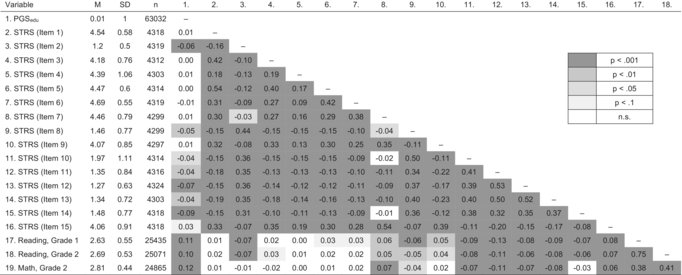

STRS items 1, 3–7, 9, and 15 are from the closeness subscale. STRS items 2, 8, and 10–14 are from the conflict subscale. Items 1, 2, 9, and 11 were used to measure positivity of teacher responses. *M*, mean; PGS_edu_, educational attainment polygenic score; *SD*, standard deviation; STRS, Student–Teacher Relationship Scale.

### Genotyping, quality control, and imputation

Genotyping, quality control, and imputation of MoBa genetic data are described by Corfield et al. (2022). After standard pre‐imputation quality control, genotypes for individuals of European genetic ancestry were imputed using the Haplotype Reference Consortium (HRC) panel (McCarthy et al., 2016). Analyses were restricted to participants of European genetic ancestry, consistent with the ancestry composition of the discovery GWAS used to construct the polygenic scores and to reduce bias due to population stratification. We then created a subsample of unrelated parent‐offspring trios with at least one genotyped member (161,566 individuals [63,032 children, 58,778 mothers, and 39,756 fathers])—see Appendix S1. The main analyses were run on the 63,032 children in this subsample.

### Measures

#### Education polygenic scores

Children's genetic propensity for educational attainment was measured using education polygenic scores (PGS_edu_) constructed using PRS‐CS (Ge, Chen, Ni, Feng, & Smoller, 2019) and summary statistics (not including 23andMe) from the Okbay et al. (2022) education genome‐wide association study (GWAS; see Appendix S2). PGS_edu_ were standardized (*M* = 0, *SD* = 1) and adjusted for sex registered at birth, genotyping plate, and the first five principal components.

#### Student–teacher relationship

Using confirmatory factor analysis (CFA), we constructed three latent variables outlined below and in Appendix S3 from the 15‐item teacher‐reported Student–Teacher Relationship Scale (STRS; Hamre & Pianta, 2001) at age 5. Items were scored on a 5‐point scale from ‘1—Never’ to ‘5—Very Often’. The full list of STRS items (and which are included in each subscale) is provided in Table S1.

##### Closeness

Constructed using the eight STRS Closeness subscale items, assessing warmth, affection, and open communication in the student–teacher relationship. These items were completed by teachers of 4,319 children. Reliability was good (α = .77) in our sample.

##### Conflict

Constructed using the seven STRS Conflict subscale items, measuring conflict and negativity in the student‐teacher relationship; higher scores represent greater conflict. These items were completed for 4,324 children. Reliability was good (α = .82). [Corrections added on 22 July 2026, after first online publication: In the preceding section, the section header has been corrected from “Reduced conflict” to “Conflict” and “reverse‐scored so higher scores represented lower conflict” has been corrected to “higher scores represent greater conflict”, in this version.]

##### Positivity of teacher responses

As Closeness and Conflict subscales include some items explicitly referring to the child's actions or affect (e.g., ‘This child easily becomes angry with me’, ‘The child openly shares his/her feelings and experiences with me’), they may partly index child behavior rather than ‘pure’ dyadic processes, meaning associations with children's genetics could partly reflect genetically influenced child traits (e.g., temperament or externalizing behavior) rather than teachers' responses per se. As we were particularly interested in whether children's genetics evoked responses from teachers, we created a preregistered variable using four items that most explicitly captured dyadic processes and teacher responses to the child: (1) ‘I share an affectionate, warm relationship with this child’; (2) ‘This child and I always seem to be struggling with each other’; (3) ‘It is easy to be in tune in with what this child is feeling’; (4) ‘Dealing with this child drains my energy’. Items 2 and 4 were reverse‐scored, so all items were in the same (positive) direction. Items were available for 4,319 children. Reliability was acceptable (α = .53).

#### Educational performance

Using CFA, we constructed a latent variable from three mother‐reported items at age 8 on children's national exam performance in: (1) reading in first grade, (2) reading in second grade, (3) arithmetic in second grade. Items were scored on a 3‐point scale: ‘1—Teacher is concerned’, ‘2—Must work more but teacher is not concerned’, ‘3—Has mastered subject well’. Reliability was good (α = .77).

### Statistical analyses

Analyses were preregistered on the Open Science Framework (Austerberry, Brandlistuen, et al., 2024). We tested our hypotheses using structural equation models (SEMs; outlined in Appendix S4) estimated in lavaan version 0.6‐10 (Rosseel, 2012), using R 4.0.3 (R Core Team, 2020), with *p* < .05 as the statistical significance threshold. There were substantially fewer data available for the STRS variables (due to the fact that childcare center questionnaires were only sent out to the last birth cohorts in MoBa, born 2007–2009) and the educational performance outcome variable than the polygenic predictor (see Table 1). Missing phenotypic data were handled using full information maximum likelihood (FIML). Details of missing data patterns, assumptions, and sensitivity analyses comparing participants with and without STRS data are reported in Appendix S5 and Table S2. Mediation was estimated using 1,000 bootstraps (Bollen & Stine, 1990). In accordance with the guidelines by Hu and Bentler (1999), we considered model fit adequate if the standardized root mean square residual (SRMR) was < .09 and the root mean square error of approximation (RMSEA) was < .06. To control for potential genetic nurture effects, we also conducted sensitivity analyses that were not preregistered, controlling for parental PGS_edu_ (see Appendix S6 and Figure S1).

## Results

### Descriptive statistics

The descriptive statistics are presented in Table 1. Mean teacher reports on items from the Closeness subscale of the STRS ranged from 4.06 to 4.69 (on a Likert scale from 1 to 5), indicating that teachers tended to view statements about student–teacher closeness as between ‘quite true’ (4) and ‘very true’ (5). Mean teacher reports on items from the Conflict subscale of the STRS ranged from 1.20 to 1.97, indicating that they viewed statements about conflict as between ‘not true at all’ (1) and ‘not quite true’ (2) on a Likert scale from 1 to 5. Mean maternal reports of children's performance in reading and math exams ranged from 2.63 to 2.81 (on a Likert scale from 1 to 3), indicating that mothers tended to rate their children as between ‘must work more but teacher is not concerned’ (2) and ‘has mastered the subject well’ (3).

### Structural equation models

**Figure 1:**
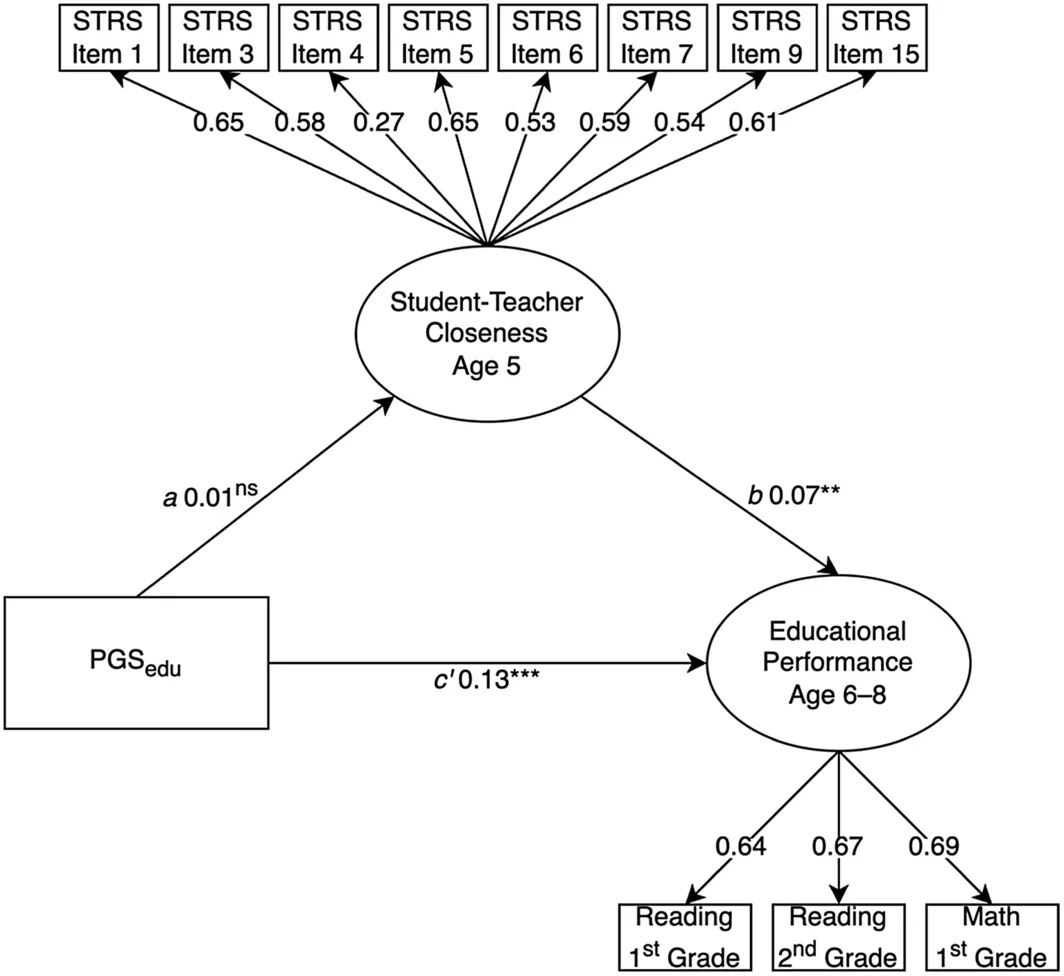
Results from structural equation models estimating the direct effects of PGS_edu_ on educational performance and indirect effects via student–teacher closeness. STRS, Student–Teacher Relationship Scale. Model fit: χ^2^(52) = 1,270.01, *p* < .001, CFI = .96, RMSEA = .02, SRMR = .04

**Figure 2:**
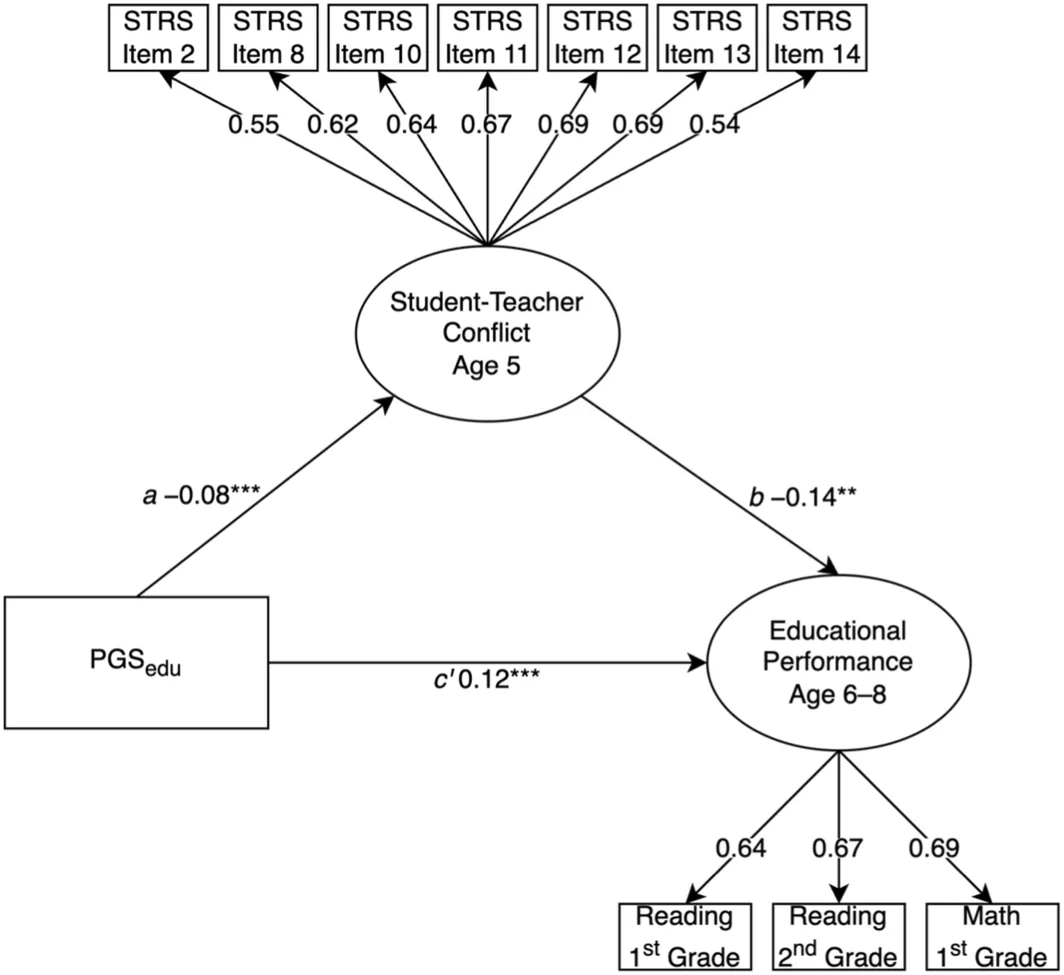
Results from structural equation models estimating the direct effects of PGS_edu_ on educational performance and indirect effects via student–teacher conflict. STRS, Student–Teacher Relationship Scale. Model fit: χ^2^(42) = 716.02, *p* < .001, CFI = .98, RMSEA = .02, SRMR = .03

**Figure 3:**
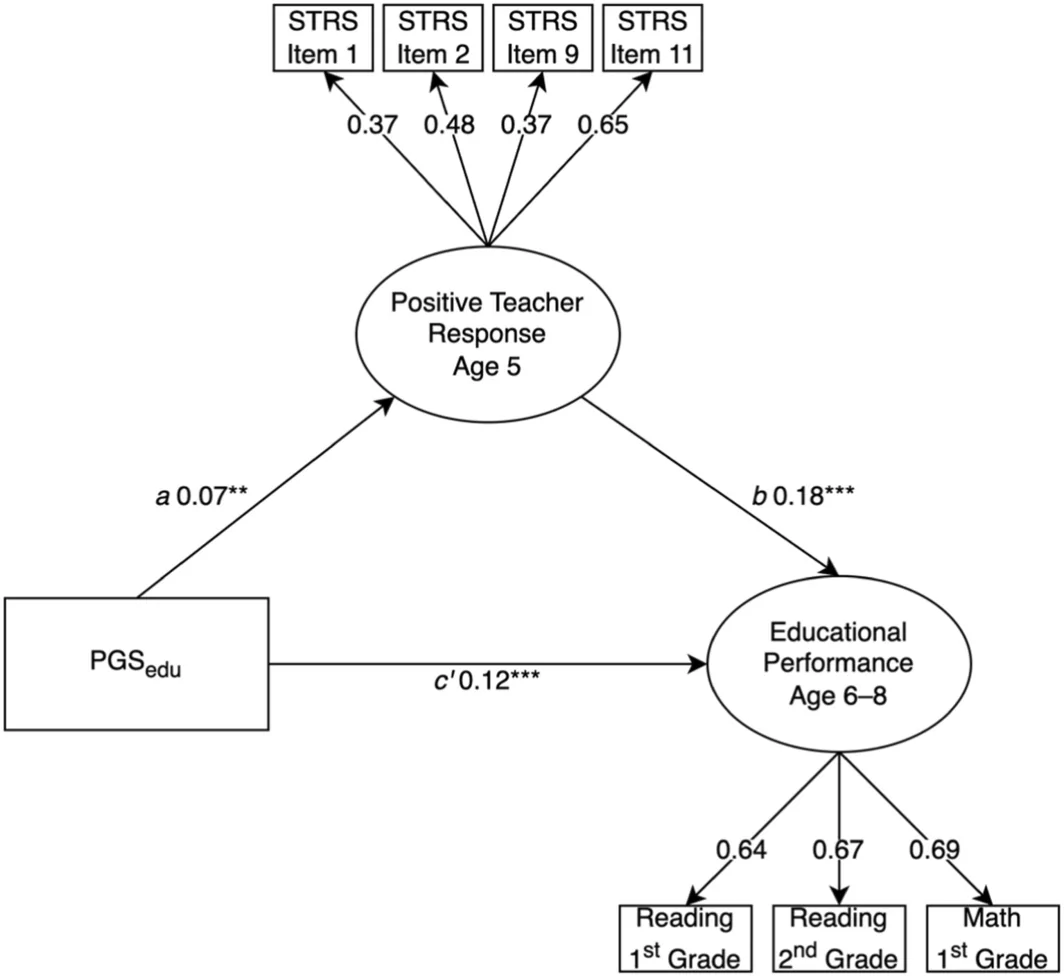
Results from structural equation models estimating the direct effects of PGS_edu_ on educational performance and indirect effects Via positive teacher responses to child. STRS, Student–Teacher Relationship Scale. Model fit: χ^2^(18) = 458.49, *p* < .001, CFI = .98, RMSEA = .02, SRMR = .04

Results from the three structural equation models (SEMs) are reported in Figures 1, 2, 3 and Table 2. The three models each had a good fit according to the preregistered thresholds (SRMR < .09, RMSEA < .06). For full model fit results, see the notes below Table 2 and Figures 1, 2, 3. The model of student–teacher closeness (Figure 1) explained 2.1% of the variance in children's educational performance at ages 6–8, the model of student–teacher conflict (Figure 2) explained 3.6% of the variance in children's educational performance, and the model of positivity of teacher responses to the child (Figure 3) explained 4.6% of the variance in children's educational performance at ages 6–8. The direct associations from these models are reported below, followed by the indirect effects via the three student–teacher relationship dimensions. Results from the sensitivity analyses controlling for parental PGS_edu_ are reported in Appendix S6.

**Table 2:** Results from structural equation models: direct, indirect, and total effects

Model	Effect	β	95% CI		*p*
			Lower	Upper	
Model 1 (Figure 1a, Closeness)	Path *a*	0.01	−0.03	0.04	.616
	Path *b*	0.07	0.03	0.11	.001
	Direct effect: *c'*	0.13	0.11	0.14	<.001
	Indirect effect: *a* × *b*	1 × 10^−3^	−2 × 10^−3^	3 × 10^−3^	.619
	Total effect: (*a* × *b*) + *c'*	0.13	0.11	0.14	<.001
Model 2 (Figure 1b, Conflict)	Path *a*	−0.08	−0.12	−0.05	<.001
	Path *b*	−0.14	−0.18	−0.10	<.001
	Direct effect: *c'*	0.12	0.10	0.13	<.001
	Indirect effect: *a* × *b*	0.01	0.01	0.02	<.001
	Total effect: (*a* × *b*) + *c'*	0.13	0.11	0.14	<.001
Model 3 (Figure 1c, Positivity of teacher responses)	Path *a*	0.07	0.03	0.11	<.001
	Path *b*	0.18	0.13	0.22	<.001
	Direct effect: *c'*	0.12	0.10	0.13	<.001
	Indirect effect: *a* × *b*	0.01	4 × 10^−3^	0.02	.019
	Total effect: (*a* × *b*) + *c'*	0.13	0.11	0.14	<.001

Model 1 (Closeness) fit: χ^2^(52) = 1,270.01, *p* < .001, comparative fit index (CFI) = .96, root mean square error of approximation (RMSEA) = .02, standardized root mean square residual (SRMR) = .04. Model 2 (Conflict) fit: χ^2^(42) = 716.02, *p* < .001, CFI = .98, RMSEA = .02, SRMR = .03. Model 3 (Positivity of teacher responses) fit: χ^2^(18) = 458.49, *p* < .001, CFI = .98, RMSEA = .02, SRMR = .04.

#### Hypothesis 1: Children's PGS_edu_ associated with student–teacher relationship

As displayed in Figure 1 and Table 2, children's PGS_edu_ were not significantly directly associated with student–teacher closeness at age 5 (*a*: β = 0.01, 95% CI [−0.03, 0.04], *p* = .616). As shown in Figures 2 and 3 and Table 2, children's PGS_edu_ were significantly directly associated with lower levels of student–teacher conflict at age 5 (*a*: β = −0.08, 95% CI [−0.12, −0.05], *p* < .001) and more positive teacher responses to students (*a*: β = 0.07, 95% CI [0.03, 0.11], *p* = .001).

#### Hypothesis 2: Student–teacher relationship associated with educational performance

As displayed in Figures 1, 2, 3 and Table 2, higher ratings of student–teacher closeness at age 5 were significantly directly associated with higher educational performance at 6–8 years (*b*: β = 0.07, 95% CI [0.03, 0.11], *p* = .001), as were lower levels of student–teacher conflict (*b*: β = −0.14, 95% CI [−0.18, −0.10], *p* < .001), and more positive teacher responses to students (*b*: β = 0.18, 95% CI [0.13, 0.22], *p* < .001).

#### PGS_edu_ associated with educational performance

As displayed in Figures 1, 2, 3 and Table 2, the direct effect of PGS_edu_ on educational performance was statistically significant in the model including student–teacher closeness (*c*′: β = 0.13, 95% CI [0.11, 0.14], *p* < .001), as well as the models on student–teacher conflict (*c*′: β = 0.12, 95% CI [0.10, 0.13], *p* < .001) and positivity of teacher responses (*c*′: β = 0.12, 95% CI [0.10, 0.13], *p* < .001). The total association between children's PGS_edu_ and educational performance at 6–8 was β = 0.13 (95% CI [0.11, 0.14], *p* < .001), explaining 2% of the variance, consistent with previous analyses of this sample (Austerberry et al., 2025).

#### Hypothesis 3: Mediation of PGS_edu_ effects via the student–teacher relationship

As displayed in Figure 1 and Table 2, student–teacher closeness when children were age 5 did not significantly mediate the association between PGS_edu_ and educational performance at ages 6–8 (a*b: β = 1 × 10^−3^, 95% CI [−2 × 10^−3^, 3 × 10^−3^], *p* = .619). As shown in Figure 2 and Table 2, reduced student–teacher conflict significantly partially mediated the effects of PGS_edu_ (a*b: β = 0.01, 95% CI [0.01, 0.02], *p* < .001), representing 10% of the total effect of the PGS_edu_ on educational performance at ages 6–8. As shown in Figure 3 and Table 2, positive teacher responses to students also significantly partially mediated the effects of PGS_edu_ (a*b: β = 0.01, 95% CI [4 × 10^−3^, 0.02], *p* = .019), representing 10% of the total effect.

## Discussion

We examined whether the student–teacher relationship mediated children's education‐linked genetic propensities. We found that the association between children's PGS_edu_ and educational performance at 6–8 years was partially mediated via student–teacher conflict and positive teacher responses to the child, but not student–teacher closeness. These results offer partial support for our hypotheses, suggesting that conflict and positive teacher responses may be implicated in the pathways linking genetic influences with educational outcomes.

In support of hypothesis 1 (H1), we found that children's PGS_edu_ were significantly associated with lower conflict and more positive teacher responses at age 5, suggesting that behaviors associated with higher PGS_edu_ may evoke reduced conflict and more positive responses. This is consistent with a meta‐analysis reporting less student–teacher conflict among more academically able students (Nurmi, 2012). Our findings also complement evidence that children's education‐associated genetics may evoke positive and cognitively stimulating caregiving at home (Austerberry et al., 2025; Austerberry, Fearon, et al., 2024; Tucker‐Drob & Harden, 2012), extending this evidence to the school context. However, contrary to H1, children's PGS_edu_ were not significantly associated with closeness. This aligns with MoBa analyses of parenting, which found children's education‐associated genetics appeared to evoke differences in maternal cognitive stimulation but not warmth (Austerberry et al., 2025). It suggests that while education‐associated genetics can evoke certain caregiver behaviors, warmth and closeness may be less easily influenced. This could reflect the relatively stable and caregiver‐driven nature of warmth and closeness, potentially making these dimensions less responsive to variation in child traits that are associated with education‐linked genetics. Alternatively, these relational qualities may be more strongly evoked by other child characteristics such as temperament and affect. Taken together, these findings highlight which aspects of the caregiving and school environments appear to be susceptible to evocative *r*GE and thus potentially suitable targets for intervention. Specifically, they point to student–teacher conflict as being a potentially more promising target than parental/teacher warmth/closeness.

Supporting hypothesis 2 (H2), all three student–teacher relationship dimensions were significantly associated with educational performance at 6–8 years, in the expected direction—higher closeness, reduced conflict, and more positive teacher responses were associated with higher performance. These findings align with extensive research linking positive student–teacher relationships with improved cognitive and educational outcomes (Roorda et al., 2011; Tao et al., 2022; Vandenbroucke et al., 2017), including previous studies from MoBa and across diverse child risk groups (Baardstu et al., 2022; Haag et al., 2025).

Finally, we found that reduced student–teacher conflict and more positive teacher responses significantly partially mediated the association between children's PGS_edu_ and their educational performance, supporting hypothesis 3 (H3). This suggests that these potentially evoked aspects of the student–teacher relationship may represent one pathway—albeit a small one—through which genetic differences are associated with educational outcomes. Student–teacher closeness did not significantly mediate the association, echoing previous null findings for caregiver warmth and closeness. This suggests these affective dimensions may be relatively insulated from children's heritable educational traits. Prior MoBa work has shown that genetic associations with achievement vary across schools (Cheesman, Borgen, et al., 2022; Cheesman, Eilertsen, et al., 2022). Our findings raise the possibility that student–teacher relationship processes represent one proximal mechanism through which such school‐level moderation may operate, although this was not tested directly here.

### Limitations and future directions

There are some key methodological limitations to consider. PGS_edu_ do not index a narrow construct of early academic ability. Rather, they capture a highly pleiotropic aggregate of genetic influences linked to educational attainment (e.g., cognition, personality, and behavior). Many of these traits likely shape classroom behavior and how teachers perceive and respond to students. Thus, observed associations should be interpreted as reflecting a constellation of genetically influenced characteristics that may evoke differential teacher responses, rather than solely an effect of early academic skills. PGS_edu_ explain only a fraction of single‐nucleotide polymorphism (SNP)‐based heritability estimates (i.e., variance explained by measured genetic variants) and twin study heritability estimates, and even less for related traits in children, thus representing a partial proxy for the genetic signal. In our sample, the PGS_edu_ explained 2% of the variance in children's educational performance and 11% of the variance in parental educational attainment (Austerberry et al., 2025), compared to twin heritability estimates of 40%–56% (Branigan, McCallum, & Freese, 2013; Silventoinen et al., 2020; Wolfram & Morris, 2023). Although PGS for educational attainment can explain more variance in adult outcomes, predictive power is typically lower in childhood and when using proxy measures of achievement. This likely reflects both developmental factors—where genetic influences on educational performance increase with age—and differences in phenotype, as our outcomes capture early educational performance rather than years of completed educational attainment. Thus, the 2% variance explained observed here is in line with expectations for this phenotype. PGS are also measured with error, due to sampling error in the discovery GWAS, imperfect tagging of causal variants (because of linkage disequilibrium), and measurement differences between the discovery and target samples. Furthermore, the GWAS we used to construct our PGS_edu_ was based on the phenotype ‘years of education by adulthood’, an indirect proxy for the early educational abilities or behaviors that may shape student–teacher relationships. Consequently, our observed associations are bounded by the predictive limits of the PGS and likely underestimate the true associations between children's latent education‐linked genetic propensities and teacher relationships.

At the same time as being underestimated, PGS associations can also be inflated by residual population stratification, unmeasured environmental confounders, and pleiotropy (whereby a PGS indexes a constellation of genetically correlated traits). Mediation estimates may also be biased; if an unmeasured confounder influences both the mediator and the outcome, it can induce a spurious mediator–outcome association that is incorrectly attributed to the mediator (Fritz, Kenny, & MacKinnon, 2016; Judd & Kenny, 1981). For instance, if genetic influences not captured by the PGS_edu_ were positively associated with both the mediator (student–teacher relationship) and the outcome (educational performance), mediation may have been overestimated. Because biases can operate in opposite directions, our results should be interpreted as observed associations between the measured PGS_edu_ and outcomes, rather than precise effect estimates. True effects may be larger or smaller depending on the relative contributions of these biases.

Phenotypic measures were also somewhat limited. Our teacher‐reported measure of the student–teacher relationship may partly index child behavior rather than ‘pure’ dyadic processes. Consequently, associations with children's PGS_edu_ may partly reflect genetically influenced child characteristics rather than solely evocative effects on teachers. Conflict and Closeness subscales include items explicitly referring to the child's actions or affect (e.g., ‘This child easily becomes angry with me’), rather than the teacher's own responses. Consequently, associations between children's PGS_edu_ and Conflict/Closeness may partly capture an association between the child's genetics and their traits (e.g., externalizing and temperament), rather than evocative effects of children's education‐linked genetics on their teachers. To address this possibility, we performed additional preregistered analyses on four items from the STRS that most explicitly captured dyadic processes and the teacher's responses to the child (e.g., ‘Dealing with this child drains my energy’). This narrower dyadic composite was still significantly associated with PGS_edu_ and mediated the same proportion of the total effect as Conflict (10%), suggesting that at least part of the signal reflects dyadic processes such as evocative *r*GE. Future work should incorporate multi‐informant or observational measures of teacher behavior to better clarify whether, and how strongly, teachers respond differently to children depending on their genetic propensities.

Children's educational performance was reported by mothers rather than directly assessed and is thus vulnerable to potential rater bias (e.g., social desirability bias). Previous MoBa analyses have uncovered different results when analyzing national test scores compared to maternal reports of children's performance (Havdahl et al., 2023). Specifically, analyses using maternal reports of children's academic performance showed weaker, less precise, and more mixed results than analyses using national test scores, with within‐family Mendelian randomization estimates generally consistent with the null (Havdahl et al., 2023). This pattern likely reflects both smaller sample sizes and the noisier, less objective nature of maternal reports compared to standardized test scores. Measurement error in maternal reports may therefore attenuate genetic associations, which helps contextualize the relatively small effect sizes observed in the present study and highlights the importance of replicating these analyses using linked national exam data not available within the present analytic dataset. At the same time, it is important to consider that maternal reports may capture broader, ecologically valid aspects of children's everyday academic functioning that may not be fully reflected in standardized test scores and are widely used within MoBa.

An additional limitation is the restricted variability in maternal‐reported educational performance and teacher‐reported student–teacher relationship. Teachers reported generally high closeness and low conflict, and mothers tended to rate children's performance positively, resulting in limited spread in the observed scores. Such range restrictions may attenuate associations and reduce statistical power, potentially contributing to the modest effect sizes observed in this study. This pattern may reflect the developmental stage assessed and the relatively high‐functioning and not fully representative nature of the population‐based sample. Future research using more diverse samples and more sensitive or objective measures may provide greater variability and more precise estimates.

Our results should be interpreted within their context. The study captured student–teacher relationships only at age 5 years and thus did not provide any evidence on whether PGS_edu_ effects vary across development. The sample was also based in a single high‐income country—Norway—and analyses suggest that, as is typical for longitudinal cohort studies, participants who remained cohort members across multiple timepoints tended to have a higher level of education than those lost to follow‐up (Vejrup, Magnus, & Magnus, 2022). Student–teacher relationship data were available only for later birth cohorts, and participants with these data were somewhat more socioeconomically advantaged and had higher PGS_edu_, indicating some selection that may limit generalizability and introduce bias, although outcome differences were small. Given this is the first test of our hypotheses, it is unclear whether the results would generalize to lower SES populations and those from varying cultural contexts and with different teacher training and educational systems. Wider research suggests SES and education equality moderate associations between genetics and cognitive/educational outcomes (Asbury & Plomin, 2013; Capron & Duyme, 1989; Tucker‐Drob & Bates, 2015; Turkheimer, Haley, Waldron, D'Onofrio, & Gottesman, 2003). Specifically, genetic effects appear to be larger in relatively uniform, high‐SES educational contexts compared to less equal, lower‐SES ones. While comparisons of MoBa with national birth registry data show that, although participants somewhat differ from the broader population, exposure–outcome associations appear largely unbiased (Nilsen et al., 2009), replication across diverse educational and cultural contexts is nonetheless needed.

Analyses were restricted to participants whose genetic data indicated European ancestry, reflecting the discovery GWAS. Most GWAS to date have been conducted in European ancestry samples (Peterson et al., 2019), limiting their broader applicability. PGS based on these European ancestry GWAS tend to have reduced predictive accuracy in other ancestral groups, though efforts are underway to improve portability and accuracy across more diverse populations (Wang, Tsuo, Kanai, Neale, & Martin, 2022). Future research should test our hypotheses in cohorts representing different ancestries. Furthermore, focusing on one ancestry does not remove the possibility of bias due to population stratification. Even when controlling for principal components, subtle residual stratification can inflate associations. Future work should prioritize generating larger within‐family GWAS to generate less‐confounded PGS, refine statistical approaches to better account for subtle population structure, and increase the ancestral diversity of GWAS.

## Conclusions

This study addressed a gap in our understanding of whether genetic effects on educational outcomes are mediated via the student–teacher relationship. Our findings indicate that teachers may respond differently to children based on their genetic propensities. Children with higher education‐linked genetic propensities tended to experience less conflict with and more positive responses from teachers. These relationship dimensions partially mediated the association between genetic propensities and later educational outcomes. This suggests that student–teacher conflict may be one potentially modifiable mechanism through which a small proportion of genetic differences influences educational success.

## Ethical considerations

The establishment of MoBa and initial data collection was based on a license from the Norwegian Data protection agency and approval from the Regional Committees for Medical and Health Research Ethics (REK). The MoBa cohort is based on regulations of the Norwegian Health Registry Act. All participating mothers and fathers provided informed written consent. The current study was approved by the REK (2020, #21076).

**Key points**

**What's known?**

- Educational attainment is heritable, but the mechanisms linking children's genetics to educational outcomes remain unclear. Student–teacher relationships are a promising yet underexplored factor that may mediate these effects.

**What's new?**

- This study provides novel evidence that student–teacher relationships can mediate genetic influences on children's educational outcomes. Children's education‐linked genetic propensities were associated with less conflict and more positive responses from teachers, which in turn partially mediated genetic effects on later educational performance.

**What's relevant?**

- These results suggest that the student–teacher relationship—in particular student–teacher conflict—may represent a pathway through which a small proportion of genetic differences are associated with educational outcomes.

## Supporting information


**Appendix S1.** Selection of a subsample of children from unrelated parent–offspring trios.
**Appendix S2.** Polygenic score construction.
**Appendix S3.** Student–Teacher Relationship Scale (STRS) items used in the study.
**Table S1.** STRS items and subscales.
**Appendix S4.** Structural equation models.
**Appendix S5.** Missing data.
**Table S2.** Comparison of participants with and without STRS data.
**Appendix S6.** Sensitivity analysis.
**Figure S1.** Structure of models adjusting for parental PGS_edu_.

## Data Availability

Data from MoBa is managed by the Norwegian Institute of Public Health. Access requires approval from the Regional Committees for Medical and Health Research Ethics (REC), compliance with GDPR, and data owner approval. Participant consent does not allow individual‐level data storage in repositories or journals. Researchers seeking access for replication must apply via www.helsedata.no.
