# Supplementary material for: Student–teacher relationships as a mediator of children's education‐linked genetics: results from the Norwegian Mother, Father and Child Cohort Study
Source: J Child Psychol Psychiatry. 2026 May 28;67(10):1729–40. doi: 10.1111/jcpp.70179 (PMC13535760; doi:10.1111/jcpp.70179)
Supplement: Supplementary file 1 [file JCPP-67-1729-s001.docx]

Student–teacher relationships as a mediator of children’s education-linked genetics: Results from the Norwegian Mother, Father and Child Cohort Study

**Supporting Information**

**Appendix S1: Selection of a Subsample of Children from Unrelated Parent–Offspring Trios**

For this study, we derived a subset of parent–offspring trios where post-quality-control (QC) genotype data were available for at least one member of the trio. Because the Norwegian Mother, Father and Child Cohort Study (MoBa) contains substantial relatedness among parents, we applied additional post-imputation QC steps beyond those described by Corfield et al. (2022) to ensure that all trios were unrelated. We also conducted steps to reduce potential batch effects, as the genotyping and imputation in MoBa were performed in multiple batches (Corfield et al., 2022).

To address relatedness and ensure that all children in our main analyses were unrelated to other parent–offspring trios in the study, we identified all pairs of individuals in the dataset (excluding those belonging to the same trio) with estimated relatedness greater than 15% (PI_HAT > 0.15 in PLINK 1.9) (Purcell et al., 2007). From each identified pair, we retained the child wherever possible, followed by the mother, then the father. In families with multiple children, one or more siblings were randomly excluded to retain only one child per trio.

To mitigate potential batch effects, we excluded single nucleotide polymorphisms (SNPs) whose minor allele frequencies (MAFs) differed significantly between genotyping batches (*p* < 1.00 x 10**^−^**^3^). Finally, we conducted a principal components analysis (PCA) in PLINK 1.9 (Purcell et al., 2007) on the final subsample to identify and control for any remaining technical artifacts.

**Appendix S2: Polygenic Score Construction**

We constructed education polygenic scores (PGS_edu_) using genome-wide association study (GWAS) summary statistics for years of education from Okbay et al. (2022), excluding 23andMe data (hereafter referred to as EA4), and the European-ancestry linkage disequilibrium (LD) reference panel from the 1000 Genomes Project Phase 3 (McVean et al., 2012). The EA4 GWAS included a small number of MoBa participants (n = 622 mothers). Because the publicly available summary statistics do not permit exclusion of individual contributing cohorts, it was not possible to remove these participants from the discovery GWAS used to construct the PGS_edu_. However, the present analytic sample consisted of children from MoBa rather than these mothers (with the exception of the sensitivity analyses reported in Appendix S6), and the overlap therefore represents only a very small proportion of the discovery sample (622 of approximately 800,000 individuals, excluding 23andMe; ~0.08%). Any inflation in predictive performance due to sample overlap is therefore expected to be negligible.

First, we retained only SNPs present in both the EA4 summary statistics and the LD reference panel. Second, we applied the QC pipeline described by Choi, Mak, and O’Reilly (2020), excluding variants that:

- were not SNPs,
- were strand ambiguous,
- had imputation INFO score < 0.90,
- had MAF < 1% in either EA4 or the LD reference panel,
- had MAF difference > 20% between EA4 and the LD reference panel,
- showed allele mismatches between EA4 and the LD reference panel,
- had out-of-bounds *p*-values (p < 0 or p > 1), or
- had duplicated IDs.

Third, we used PRS-CS to apply Bayesian shrinkage to SNP effect sizes, with the following parameters:

- 25,000 MCMC iterations,
- 10,000 burn-in,
- global shrinkage = 0.01,
- gamma-gamma prior parameters a = 1 and b = 0.5, and
- thinning factor = 5.

Fourth, the SNPs in our subsample were subjected to the same QC steps as applied to the EA4 summary statistics. Finally, the PGS_edu_ were calculated in PLINK 1.9 using the PRS-CS–adjusted effect sizes. After QC, 502,729 SNPs remained common across EA4, MoBa, and the LD reference panel. PGS_edu_ were then constructed for 161,566 individuals (63,032 children, 58,778 mothers, 39,756 fathers). The PGS_edu_ from the 63,032 children in this subsample were used for the present analyses.

Before testing our hypotheses, PGS_edu_ were standardized (M = 0, SD = 1) and residualized by regressing on sex, genotyping plate (to control for plate-specific effects, which have been detected in the MoBa sample) and the first 5 PCs derived from a PCA of our subsample. Prior to this adjustment, we conducted sensitivity checks on height data including adjustment for the first 10 PCs. Results were substantively unchanged compared to models including five PCs. Because the structural equation models in this study were relatively complex, we retained five PCs in the final models to limit the number of covariates while still accounting for residual population structure.

To evaluate the performance of the PGS_edu_, prior to the main analyses, we regressed the parental PGS_edu_ on self-reported parental educational attainment collected at 15 weeks’ gestation. Maternal PGS_edu_ explained 10.52% of the variance in maternal education (*p* < .001), and paternal PGS_edu_ explained 11.28% of the variance in paternal education (*p* < .001). Effect sizes were positive, as expected, and variance explained was slightly lower than the 12–16% reported by Okbay et al. (2022), who included 23andMe data in their GWAS.

As a negative control, we regressed mother, father, and child residualized PGS_edu_ on child height, reported by mothers when their children were 8 years old. As expected, none of the associations were significant (*p* > .05), and all explained < 0.01% of variance.

**Appendix S3: Student–Teacher Relationship Scale (STRS) Items Used in the Study**

The student–teacher relationship variables were derived from the teacher-reported Student–Teacher Relationship Scale (STRS; Pianta, 2001) administered when children were 5 years old. Teachers rated each item on a 5-point scale ranging from 1 (“Never”) to 5 (“Very often”).

Items were grouped into the Closeness and Conflict subscales following the standard STRS structure. In addition, a preregistered composite (Positivity of Teacher Responses) was constructed using four items that most directly reflected teachers’ responses within the dyadic relationship. Items referring to negative interactions were reverse scored when constructing variables so that higher values indicated more positive teacher responses.

The items used in this study and their assignment to constructs are listed in Table S1.

**Table S1.** STRS Items and Subscales

| **Item** | **Subscale** | **Positivity composite** |
| --- | --- | --- |
| 1. I share an affectionate, warm relationship with this child | Closeness | Yes |
| 2. This child and I always seem to be struggling with each other | Conflict | Yes |
| 3. If upset, this child will seek comfort from me | Closeness | No |
| 4. This child is uncomfortable with physical affection or touch from me | Closeness | No |
| 5. This child values his/her relationship with me | Closeness | No |
| 6. When I praise this child, he/she beams with pride | Closeness | No |
| 7. This child spontaneously shares information about himself/herself | Closeness | No |
| 8. This child easily becomes angry with me | Conflict | No |
| 9. It is easy to be in tune with what this child is feeling | Closeness | Yes |
| 10. This child remains angry or is resistant after being disciplined | Conflict | No |
| 11. Dealing with this child drains my energy | Conflict | Yes |
| 12. When the child is in a bad mood, I know we're in for a long and difficult day | Conflict | No |
| 13. This child's feelings about me can be unpredictable or can change suddenly | Conflict | No |
| 14. This child is sneaky and manipulative with me | Conflict | No |
| 15. The child openly shares his/her feelings and experiences with me | Closeness | No |

**Appendix S4: Structural Equation Models**

We tested the hypothesized mediation by running three separate structural equation models (SEMs) in lavaan version 0.6-10 (Rosseel, 2012), R 4.0.3 (R Core Team, 2020)—one for each of the hypothesized mediators (closeness, conflict, and positivity of teacher responses). Each model included three variables: (1) the predictor, PGS_edu_; (2) a latent variable measuring one of the three hypothesized mediators at age 5; (3) a latent variable measuring the outcome, child educational performance at age 8. Paths were specified from the predictor to the mediator, from the mediator to the outcome, and directly from the predictor to the outcome, allowing for the estimation of both direct and indirect (i.e., mediated) effects of the PGS_edu_.

**Appendix S5: Missing Data**

We addressed missing data using full information maximum likelihood (FIML), a method shown in simulation studies to produce unbiased parameter estimates and standard errors under conditions of data missing completely at random (MCAR) or missing at random (MAR) and to perform better than listwise deletion (Enders & Bandalos, 2001).

Little's MCAR test, conducted in R version 4.0.3 (R Core Team, 2020) using the *naniar* package version 0.6.1 (Tierney, Cook, McBain, & Fay, 2021), indicated that the data were not MCAR: χ^2^ (9,302) = 11,543, *p* < .001. Under MCAR, missingness occurs entirely at random and is unrelated to any observed or unobserved variables.

Although we could not definitively exclude the possibility of data being missing not at random (MNAR)—where missingness is associated with unobserved values—the Little’s test results were consistent with the MAR assumption, where missingness is associated with the observed variables but not to unobserved data. The MAR assumption is compatible with the use of FIML, which was applied throughout the analyses.

STRS data were available for a subset of participants because questionnaires to childcare centers were administered only to the later MoBa birth cohorts (2007–2009). As a result, missingness in STRS variables is partly design-based and cohort-linked rather than solely due to participant attrition. To assess potential selection into the STRS subsample, we compared participants with at least one observed STRS item to those missing all STRS items on key variables, including child PGSedu, child educational outcomes at 6–8 years (using the three items included in the educational attainment latent variable), and parental educational attainment (as a proxy for family SES).

Participants with STRS data differed from those without STRS data on several variables. The STRS subsample had higher parental education (mother: SMD = 0.38; father: SMD = 0.26) and higher child polygenic scores (SMD = 0.19), indicating some degree of selection. Differences in child educational performance at 6–8 years were small (SMDs = 0.06–0.07). Full results are reported in Table S2.

These differences likely reflect, at least in part, cohort/design effects, given that STRS data were only collected in later birth cohorts, rather than dropout-related attrition alone. While these patterns indicate some selectivity, the relatively small differences in outcomes and moderate differences in predictors are consistent with the plausibility of the MAR assumption underlying the FIML analyses.

**Table S2.** Comparison of participants with and without STRS data

|  | n | | M | |  |  |
| --- | --- | --- | --- | --- | --- | --- |
| Variable | No STRS | STRS | No STRS | STRS | p | SMD |
| Child PGS | 58703 | 4329 | -0.01 | 0.19 | < .001 | 0.19 |
| Child reading, grade 1 | 22028 | 3407 | 2.62 | 2.66 | < .001 | 0.07 |
| Child reading, grade 2 | 21721 | 3350 | 2.68 | 2.72 | < .001 | 0.06 |
| Child math, grade 2 | 21545 | 3320 | 2.80 | 2.82 | < .001 | 0.06 |
| Mother EA | 51920 | 4124 | 4.54 | 5.00 | < .001 | 0.38 |
| Father EA | 40728 | 3666 | 4.22 | 4.59 | < .001 | 0.26 |

**Appendix S6: Sensitivity Analysis**

To control for potential genetic nurture effects, we conducted a series of sensitivity analyses that were not preregistered, controlling for parental PGS_edu_. Figure S1 displays the structure of these models diagrammatically. In primary models, child PGS_edu_ was associated with teacher–child conflict (β = −0.08, SE = 0.027, 95% CI [−0.12, −0.05], *p* < .001) and positive teacher responses (β = 0.07, SE = 0.021, 95% CI [0.03, 0.11], *p* = .001), but not closeness. After adjusting for maternal and paternal PGS_edu_, effect sizes were modestly attenuated but remained similar in magnitude (conflict: β = −0.07, SE = 0.031, 95% CI [−0.13, −0.01], *p* = .035; positive teacher responses: β = 0.05, SE = 0.038, 95% CI [−0.03, 0.12], *p* = .213). In contrast, parental PGS_edu_ were not independently associated with student–teacher outcomes. Adjustment was accompanied by increased standard errors, consistent with high intercorrelation among child and parental genotypes and reduced effective information. Overall, inclusion of parental PGS_edu_ did not materially change the estimated associations, although precision decreased in adjusted models.

Indirect effects via teacher–child conflict and positive teacher responses were statistically significant in unadjusted models (conflict: ab = 0.01, SE = 0.003, 95% CI [0.01, 0.02], *p* < .001; evoked: ab = 0.01, SE = 4×10^−3^, 95% CI [4×10^−3^, 0.02], *p* = .003). After adjusting for parental PGS, indirect effect estimates were modestly reduced in magnitude (conflict: ab = 0.01, SE = 0.005, 95% CI [0.00, 0.02], *p* = .053; evoked: ab = 0.01, SE = 0.007, 95% CI [−0.01, 0.02], *p* = .268) and confidence intervals widened. Closeness did not mediate associations in either specification. As with the direct effects, parental adjustment primarily reduced precision rather than eliminating the mediated associations.

**Figure S1.** Structure of models adjusting for parental PGS_edu_

**References**

Choi, S. W., Mak, T. S.-H., & O’Reilly, P. F. (2020). Tutorial: A guide to performing polygenic risk score analyses. *Nature Protocols, 15*(9), 2759-2772. doi:10.1038/s41596-020-0353-1

Corfield, E., Frei, O., Shadrin, A. A., Rahman, Z., Lin, A., Athanasiu, L., . . . Havdahl, A. (2022). The Norwegian Mother, Father, and Child cohort study (MoBa) genotyping data resource: MoBaPsychGen pipeline v.1. Preprint: <https://www.biorxiv.org/content/10.1101/2022.06.23.496289v2>.

Enders, C. K., & Bandalos, D. L. (2001). The relative performance of full information maximum likelihood estimation for missing data in structural equation models. *Structural Equation Modeling: A Multidisciplinary Journal, 8*(3), 430-457. doi:10.1207/S15328007SEM0803_5

McVean, G. A., Altshuler, D. M., Durbin, R. M., Abecasis, G. R., Bentley, D. R., Chakravarti, A., . . . Ruiz-Linares, A. (2012). An integrated map of genetic variation from 1,092 human genomes. *Nature, 491*(7422), 56-65. doi:10.1038/nature11632

Okbay, A., Wu, Y., Wang, N., Jayashankar, H., Bennett, M., Nehzati, S. M., . . . LifeLines Cohort, S. (2022). Polygenic prediction of educational attainment within and between families from genome-wide association analyses in 3 million individuals. *Nature Genetics, 54*(4), 437-449. doi:10.1038/s41588-022-01016-z

Purcell, S., Neale, B., Todd-Brown, K., Thomas, L., Ferreira, M. A., Bender, D., . . . Sham, P. C. (2007). PLINK: A tool set for whole-genome association and population-based linkage analyses. *American Journal of Human Genetics, 81*(3), 559-575. doi:10.1086/519795

R Core Team. (2020). *A language and environment for statistical computing*. Vienna, Austria: R Foundation for Statistical Computing.

Rosseel, Y. (2012). lavaan: An R package for structural equation modeling. *Journal of Statistical Software, 48*(2), 1-36. doi:10.18637/jss.v048.io2

Tierney, N., Cook, D., McBain, M., & Fay, C. (2021). *naniar: Data structures, summaries, and visualisations for missing data. R package version 0.6.1.*
